# Self-esteem is associated with premorbid adjustment and positive psychotic symptoms in early psychosis

**DOI:** 10.1186/1471-244X-11-136

**Published:** 2011-08-19

**Authors:** Kristin Lie Romm, Jan Ivar Rossberg, Charlotte Fredslund Hansen, Elisabeth Haug, Ole A Andreassen, Ingrid Melle

**Affiliations:** 1 Division of Mental Health and Addiction, Oslo University Hospital, 0407 Oslo, Norway; 2 Institute of clinical medicine, Section of psychiatry, University of Oslo, 0318 Oslo, Norway; 3 Department of Psychology, University of Oslo, 0318 Oslo, Norway; 4 Department of Psychosis and Rehabilitation, Sykehuset Innlandet HF, Norway

**Keywords:** Self-esteem, First episode psychosis, Schizophrenia, Premorbid adjustment, Delusions, Hallucinations

## Abstract

**Background:**

Low levels of self-esteem have been implicated as both a cause and a consequence of severe mental disorders. The main aims of the study were to examine whether premorbid adjustment has an impact on the subject's self-esteem, and whether lowered self-esteem contributes to the development of delusions and hallucinations.

**Method:**

A total of 113 patients from the Thematically Organized Psychosis research study (TOP) were included at first treatment. The Positive and Negative Syndrome Scale (PANSS) was used to assess present symptoms. Premorbid adjustment was measured with the Premorbid Adjustment Scale (PAS) and self-esteem by the Rosenberg Self-Esteem Scale (RSES).

**Results:**

Premorbid social adjustment was significantly related to lower self-esteem and explained a significant proportion of the variance in self-esteem. Self-esteem was significantly associated with the levels of persecutory delusions and hallucinations experienced by the patient and explained a significant proportion of the variance even after adjusting for premorbid functioning and depression.

**Conclusion:**

There are reasons to suspect that premorbid functioning is an important aspect in the development of self- esteem, and, furthermore, that self-esteem is associated with the development of delusions and hallucinations.

## 1. Background

Self-esteem, a global and complex concept, is comprised of both appraisal of self-worth based on personal achievements and anticipation of evaluation by others [1,2]. Although not uniformly low, self-esteem is often found to be compromised among persons with mental illnesses [3]. Low self-esteem is therefore of considerable interest as it is both a possible consequence and a possible cause of psychiatric symptoms [4-6].

Regarding self-esteem as a consequence of mental illness, studies predictably show that stigmatization and self-stigmatization may lower self-esteem in persons with mental illness [7]. Low self-esteem also appears to increase the risk of psychiatric disorders such as depression, eating disorders and substance abuse [8]. In psychotic disorders, low self-esteem has been implicated in both the development of delusions [9,10] and the maintenance of psychotic symptoms [11].

Recent models of global self-esteem suggest that it is both a trait and a state measure [12]. People have a typical, average or trait level of self-esteem, while their momentary, or 'state', judgments of self-esteem can fluctuate around this level dependent on social feed-back and self-judgment. Furthermore, it is the person's interpretation of the event or circumstance, and its relevance to his or her contingencies of self-worth, that determines both *if *and *how strongly *it will affect state self-esteem [12,13]. It appears that treatment failures, functional loss, demoralization and stigmatization may lower self-esteem in patients with severe mental illnesses. To what extent low levels of self-esteem in severe mental disorders are based on underlying, or trait levels of self-esteem, and how this in turn may increase vulnerability to more severe symptoms has not been thoroughly explored. This is of importance both for the understanding of the mechanisms behind the development of psychotic symptoms and also for improving treatment as self-esteem can be influenced by therapeutic interventions [14,15].

Studies have suggested that difficult childhood experiences such as childhood loss and social marginalization contribute to a cognitive vulnerability accompanied by a negative view both towards the person himself and towards others [4,11,16]. It can be hypothesized that individuals with a history of poor premorbid adjustment, both social and academic, are more prone to negative self-evaluation and reduced global self-esteem. MacBeth and Gumley have shown in their review of premorbid adjustment and early symptom development that premorbid problems in psychosocial functioning are associated with a greater severity of illness course and, in particular, more negative symptoms [17]. They also found that reduced quality of life (QoL) was reported by individuals with poorer premorbid functioning. Interestingly, the course of premorbid social adjustment has been found to exert a greater effect on QoL than premorbid academic adjustment [18], and may also be more influential on trait self-esteem. This underlines the importance of separating the social and academic domains of premorbid adjustment.

To our knowledge only one study has tried to examine the relationship between premorbid adjustment and self-esteem in patients with schizophrenia spectrum disorder [19]. They found no relationships between self-esteem and premorbid adjustment in recovered psychotic patients. However, premorbid adjustment was not assessed with a specific instrument which may account for the negative results.

A relatively rich literature exists on the relationship between low self-esteem and symptom formation in severe mental disorders including psychotic disorders. One study showed that the contents of patient's delusions were consistent with patient's global self-esteem and suggested that low self-esteem accounted for the persistence of delusions [20]. Other studies found significant correlations between negative self-evaluation and a wider variety of positive symptoms i.e hallucinations and delusions, in schizophrenia [10]. It has also been found that patients with a low level of self-esteem and more depressive symptoms had more intense auditory hallucinations with a more negative content [21]. In addition, it has been found that patients who had both high levels of suspiciousness and low self-esteem made more misattributions of anger which may also fuel delusional ideation [22]. This is in line with findings from the general population, where delusion prone individuals show lower self-esteem [23]. Finally, it has been found that several delusional themes including persecution, thought disturbances/thought broadcasting, catastrophic ideation, and negative self beliefs were related to low self-esteem [24].

Other studies have shown higher levels of self-esteem in patients with delusional disorder compared to depressed patients [25]. However, the authors found that the group without depressive symptoms had significantly higher levels of grandiose ideation than the other groups which may have accounted for the elevated levels of self-esteem. The authors concluded that persecutory delusions may reflect an attributional style protecting the individual from low self-esteem. The same has been hypothesized for grandiose delusions, but the few studies in this area do not clearly support this hypothesis [26]. Other studies have found equal levels of self-esteem in patients with delusions and matched healthy controls with both groups demonstrating higher levels than depressed patients [27].

Self-esteem has been found to fluctuate over the short-term. It has been demonstrated that paranoid individuals display more fluctuations in their self-esteem, and that the fluctuation predicts the degree of subsequent increase in paranoid thinking [28]. However, other studies indicate that changes in both positive and negative beliefs about the self are related more to changes in negative symptoms than changes in paranoid symptoms [29]. In summary the relationship between premorbid function, self-esteem and the formation of psychotic symptoms remains unclear.

To date the relationship between self-esteem, psychotic symptoms and premorbid adjustment in the early stages of psychosis has not been thoroughly explored. Previous studies of that nature have all been conducted with patients with chronic psychotic disorders where the effects of a long-term severe illness and secondary processes may significantly confound relationships. More studies are thus needed to explore the relationship between self-esteem and psychotic symptoms during the early phases of psychotic disorder. This is of importance as patients coming to their first treatment for a psychotic disorder are less influenced by stigmatization, treatment failures, and subsequent disappointments which may contribute to lowered self-esteem.

The aims of the current study are thus to investigate the following questions in a large and well characterized group of patients with first episode psychosis:

1) To what extent is premorbid adjustment (as measured by the Premorbid Adjustment Scale (PAS)), related to self-esteem (as measured by the Rosenberg Self- Esteem Scale (RSES)), in this patient group?

2) To what extent is self -esteem related to the level of hallucinations and delusions (as measured by the Positive and Negative Syndrome Scale (PANSS))?

## 2. Method

### 2.1 Subjects

From February 2007 to October 2009, 113 patients from the main psychiatric treatment centres in Oslo and two neighbouring counties were consecutively included in the Thematically Organized Psychosis research study (TOP). The inclusion criteria were that they were within the age bracket of 18 to 65 years old and that they were coming to their first treatment for a schizophrenia spectrum disorder as defined in DSM-IV. Exclusion criteria were a history of organic brain disorder, a significant co-morbid medical condition or an IQ of less than 70.

The diagnostic distribution was as follows; (N (%)): schizophrenia 68 (60.2%), schizophreniform disorder 7 (6.2%), schizoaffective disorder 11 (9.7%), brief psychosis 1 (0.9%), delusional disorder 7 (6.2%) and psychosis NOS 19 (16.8%).

Patients were eligible for inclusion up to 52 weeks after the start of the first adequate treatment for their disorder and were not considered as First Episode Psychosis (FEP) patients if they had previously been treated with anti-psychotic medication in adequate dosage for more than 12 weeks, or until remission. Being psychotic was defined as having a rating of 4 or more on the PANSS items p1 (delusions), p2 (disorganisation), p3 (hallucinations), p5 (grandiosity), p6 (persecutory delusions) or (g9) (unusual thought content) for more than one week. The mean age of the patients was 25.8 (SD 7.7). 37 (32.7%) were females and 76 (67.3%) were male. 82 (72.6%) were single, 24 (21.2%) were married or co-habiting, and 7 (6.3%) were divorced, separated or widowed. Mean years of education was 12.4 (SD 2.72) and median duration of untreated psychosis (DUP) was 78 weeks (range 0-1040) (N = 106). All patients gave written informed consent and the study was approved by the regional research ethics committee.

### 2.2 Assessments

#### 2.2.1 Measures

Diagnosis was set according to the Structured Clinical Interview for Diagnostic and Structural Manual of Mental Disorders, fourth version (SCID I interview for the DSM -IV) [30]. Current severity of psychotic symptoms was measured with the Structural Clinical Interview of the Positive And Negative Syndrome Scale (SCI-PANSS) [31]. Self-esteem was measured using the Rosenberg Self-Esteem Scale (RSES) [32]. This is a 10 item self-administered questionnaire with a 4-point likert-type response set, ranging from strongly disagree to strongly agree.

Depression was diagnosed according to the criteria in DSM-IV. We only measured major depression to avoid overlap with negative symptoms. The duration of untreated psychosis (DUP) was measured according to previously published criteria [33]. Premorbid adjustment was measured with the Premorbid Adjustment Scale (PAS) [34]. The premorbid phase is defined as the time from birth until 6 months before onset of psychosis. The PAS measures both social and academic functioning during four age intervals. We only included the age range of childhood (birth -11 years) and early adolescence (12-15 years) as the peak age for the onset of schizophrenia spectrum disorders is early adulthood. We thus tried to avoid 'contaminating' the premorbid period as it can be difficult to point out the exact period of conversion to psychosis, especially in individuals with insidious onset. Information was collected with regard to each age range directly from the patient, from historical medical records and from significant family members where appropriate. From this data ratings of sociability and withdrawal, peer relationships, academic performance and adaptation to school were made.

As the current study is part of a broad research initiative with an extensive interview protocol, most participants chose to divide the interview into 2-3 sections over 1-2 weeks. Significant efforts were made to make the assessments as close in time as possible.

#### 2.2.2. Procedures

The patients were interviewed by trained psychologists and psychiatrists at the same time as the SCID-I was administered. The investigators had all completed general training and a reliability program with regard to the TOP research study. For DSM-IV diagnostics mean overall kappa with training videos was 0.77, and mean overall kappa for a randomly drawn subset of actual study patients was also 0.77 (95% CI 0.60-0.94). Inter-rater reliability, measured by the intra class correlation coefficient (ICC 1.1) was 0.82 (95% CI 0.66-0.94) for the PANSS positive subscale, 0.76 (95% CI 0.58-0.93) for the PANSS negative subscale and 0.73 (95% CI 0.54-0.90) for the PANSS general subscale.

## 3. Statistical analysis

Correlations between demographic/clinical characteristics and self-esteem were calculated using Pearson's product moment co-efficients. To estimate how much of the variance in self-esteem was explained independently by premorbid functioning we performed a block-wise hierarchical multiple regression analysis with age and gender entered in the first block and premorbid adjustment in the second block. As academic adjustment in childhood versus academic adjustment in early adolescence, and social adjustment in childhood versus social adjustment in early adolescence were strongly inter-correlated (with r = 0.66 and r = 0.77 respectively), only results for early adolescence were entered to represent PAS and avoid co-linearity problems. The associations between global self-esteem and hallucinations and delusions, both general and persecutory, were analyzed similarly using Pearson's correlations and followed up with three block-wise hierarchical multiple regression analysis with hallucinations, delusions and persecutory delusions as the dependent variables. Demographic information was placed in the first block, premorbid adjustment in the second block, depression i.e whether the patient was in a major depressive episode or not, in the third block and self-esteem in the fourth. By entering self-esteem in the fourth we adjusted for the amount of variance explained by the variables in the first three blocks. Finally, we conducted various interactional analyses to explore whether self-esteem acted as a mediator or moderator of the relationship between premorbid adjustment and symptoms.

## 4. Results

Table 1 shows the patient characteristics of the 113 included patients.

**Table 1:** Demographics, n = 113.

	Mean	SD
Age	25.79	7.7
Females (N/%)	37	33
Years of education	12.4	2.72
DUP (median/range)	78	0-1040
PANSS:		
Positive score	17.4	4.21
Negative score	16.28	6.03
General score	36.74	8.03
Total score	69.99	15.14
RSES	22.81	6.16
Current depression MDE (N/%)	24	21.24
Diagnosis (N/%)		
Schizophrenia	68	60.18
Schizophreniform disorder	7	6.19
Schizoaffective disorder	11	9.73
Delusional disorder	7	6.19
Brief psychosis	1	0.88
Psychosis NOS	19	16.81

Abbreviations:

DUP; Duration of Untreated Psychosis

PANSS; Positive and Negative Syndrome Scale

RSES; Rosenberg Self-Esteem Scale

MDE; Major Depressive Episode

NOS; Not Otherwise Specified

As shown in table 2, self-esteem was significantly correlated with several demographic and clinical characteristics, including the four sub-scale measures of premorbid adjustment and with current levels of symptoms (depression, persecutory delusions and hallucinations, poor rapport and stereotyped thinking). Furthermore, females reported lower self-esteem than men.

**Table 2:** Mean and standard deviations for patient characteristics and their correlations with RSES.

	Mean	SD	RSES
RSES	22.81	6.14	1.00
Age	25.79	7.70	0.08
Gender	1.33	0.47	-0.41**
PAS			
Childhood social	2.76	3.22	-0.27**
Childhood academic	3.96	2.93	-0.19*
Early adolescence social	3.56	3.30	-0.30**
Early adolescence academic	4.93	2.90	-0.22*
PANSS			
P1 Delusions	3.85	1.32	-0.17
P2 Disorganized	1.95	1.17	0.14
P3 Hallucination	3.24	1.65	-0.29**
P4 Excitement	1.91	1.05	-0.04
P5 Grandiosity	1.68	1.34	0.09
P6 Suspiciousness	3.33	1.50	-0.30**
P7 Hostility	1.45	0.80	-0.13
N1 Blunted affect	2.46	1.37	0.07
N2 Emotional withdrawal	2.59	1.19	0.13
N3 Poor rapport	2.22	1.27	0.24*
N4 Apathetic social withdrawal	2.83	1.46	0.14
N5 Abstract thinking	2.38	1.34	0.12
N6 Lack of flow	2.22	1.43	0.15
N7 Stereotyped thinking	1.58	0.93	0.25**
Depression MDE	1.79	0.41	0.28

**. Correlation is significant at the 0.01 level (2-tailed).

*. Correlation is significant at the 0.05 level (2-tailed).

Abbreviations:

RSES; Rosenberg Self-Esteem Scale

PAS; Premorbid Adjustment Scale

PANSS; Positive and Negative Syndrome Scale

MDE; Major Depressive Episode

In the first hierarchical multiple regression analysis, with self-esteem as the dependent variable, the included variables explained 25% of the variance in self-esteem (Table 3). Only gender and social adjustment in early adolescence contributed significantly to the level of global self-esteem. Gender explained 16% of the variance while premorbid social adjustment explained an additional 9%.

**Table 3:** Multiple hierarchical regression analysis with self-esteem as dependent variable.

Model	Unstandardized Coefficients	Standardized Coefficient s		t	**Sig**.	95% Confidence Interval for B		Adjusted R Square
	**B**	**Std.** **Error**	**Beta**			**Lower** **Bound**	**Upper** **Bound**	

Age	0.04	0.07	0.05	0.67	0.507	-0.09	0.17	
Gender	-5.35	1.07	-0.41	-5.00	0.001	-7.47	-3.23	0.16
PAS Early adolescence social	-0.43	0.17	-0.23	-2.55	0.012	-0.77	-0.10	
PAS Early adolescence academic	-0.29	0.19	-0.14	-1.51	0.133	-0.67	0.09	0.25

Explained variance for final model: R^2 ^= 0.25, F = 10.19, P < 0.001

Dependent Variable: Rosenberg self-esteem scale (RSES)

Abbreviations:

PAS; Premorbid Adjustment Scale

In the second hierarchical multiple regression analysis performed, with positive psychotic symptoms as the dependent variable, self-esteem explained a significant amount of the variance in both hallucinations and persecutory delusions, even after adjusting for age, gender, premorbid adjustment and depression. In general, levels of self esteem did not explain a significant amount of the variance in occurrence of delusions (P1) (Table 4).

**Table 4:** Multiple hierarchical regression analysis with hallucinations and persecutory delusions as dependent variables.

	Unstandardized Coefficients		Standardized Coefficients	t	**Sig**.	95% Confidence Interval for B		Adjusted R Square
	**B**	**Std. Error**	**Beta**			**Lower Bound**	**Upper Bound**	

Age	-0.05	0.02	-0.22	-2.48	0.01	-0.08	-0.01	
Gender	0.37	0.34	0.11	1.06	0.29	-0.32	1.05	0.08
PAS Early adolescence social	-0.06	0.05	-0.12	-1.18	0.24	-0.16	0.04	
PAS Early adolescence academic	0.10	0.06	0.18	1.83	0.07	-0.01	0.21	0.10
Depression MDE	-0.30	0.37	-0.08	-0.82	0.41	-1.04	0.43	0.11
RSES	-0.06	0.03	-0.21	-1.94	0.05	-0.11	0.00	0.13

a. Dependent Variable: Hallucinations (PANSS p3)								
Explained variance for final model: R2 = 0.13, F = 3.37, p = 0.002								

Age	0.03	0.02	0.14	1.63	0.11	-0.01	0.06	
Gender	0.08	0.31	0.02	0.25	0.80	-0.54	0.70	0.01
PAS Early adolescence social	0.07	0.05	0.15	1.52	0.13	-0.02	0.16	
PAS Early adolescence academic	0.09	0.05	0.17	1.71	0.09	-0.01	0.19	0.11
Depression MDE	0.26	0.34	0.07	0.78	0.44	-0.40	0.93	0.10
RSES	-0.06	0.03	-0.24	-2.25	0.03	-0.11	-0.01	0.13

a. Dependent Variable: Persecutory delusions (PANSS p6)

Explained variance for final model: R2 = 0.13, F = 3.84, p = 0.002

Abbreviations:

PAS: Premorbid Adjustment Scale

MDE; Major Depressive Episode

RSES; Rosenberg self-esteem scale

PANSS; Positive and Negative Syndrome Scale

Finally, various interactional analyses revealed no significant interaction between premorbid adjustment and symptoms mediated or moderated by self-esteem.

## 5. Discussion

This study demonstrates both a statistically significant relationship between poor premorbid social adjustment and low levels of global self-esteem and between self-esteem and positive psychotic symptoms i.e hallucinations and persecutory delusions. The relationship between self-esteem and positive psychotic symptoms remained significant even after adjusting for the presence of a major depressive episode indicating that this effect is not mediated by the presence of depressive symptoms.

The current study is one of the first to show a relationship between poor premorbid social adjustment and level of global self-esteem in psychotic disorders. The only other study exploring this relationship [19] did not apply a specific validated measure of premorbid adjustment such as the PAS, but instead divided subjects by use of data collected by means of the Diagnostic interview for Psychosis (DIP) into 'yes' or 'no' for poor premorbid adjustment. This implies both a less validated measure of premorbid adjustment and a subsequent loss of variance in statistical analysis. In addition their sample consisted of older participants with a longer duration of illness, and thus a higher risk for recall bias.

Premorbid social adjustment as a concept incorporates issues including such factors as how you interact with your schoolmates, adjust to groups and friends and the presence of age-relevant sexual interest. Previous studies have shown that general cognitive abilities, exposure to bullying, social marginalization, abuse, neglect or the presence of neurodevelopmental factors are the strongest predictors of social adjustment [35-37]. Many patients with psychotic disorder may have a premorbid vulnerability that reduces their ability to achieve and maintain social competence and thus affect their premorbid social adjustment [9,11,38]. This will in turn affect the individuals schematic beliefs about themselves and others. These beliefs influence how we experience ourselves in relation to the world. In these patients the effect on these schematic beliefs may lead to social adversity and a feeling of low self-esteem [16].

Furthermore, studies of persons with auditory hallucinations have shown that voice hearers experience a subordinate relationship to their voices mirroring other social relationships. This suggests the existence of maladaptive inter-personal schemata serving both [39]. These schemata are not necessarily a result of the psychotic illness, but may be a result of poor premorbid social adjustment and are in line with theories of how long-term experience of social defeat can be a risk factor for psychosis [35].

We also found self-esteem to be a predictor of both hallucinations and persecutory delusions in early psychosis, even though the explained variance was rather moderate. This is in line with previous studies [10,21,28,40]. Garety's cognitive model of psychosis [11] suggests that the experience of social adversity and lowered self-esteem can lead to the development of psychotic symptoms through an increased vulnerability to psychotic disorders.

We further argue that poor premorbid social adjustment with social withdrawal and subsequent marginalization provides content for psychotic attribution due to a lack of correcting social feedback. This is supported by findings in studies of patients at high risk of developing psychosis [41]. In line with this, cognitive behavioral therapy aiming to improve self-esteem by correcting misattributing tendencies has shown clinical benefits in terms of both increased self-esteem, reduced positive symptoms and improved social functioning [14].

Our findings are also supported by findings in the general population. Negative ideas about oneself and others have been found to be predictors of paranoid thinking in the general population [42]. In addition, premorbid neuroticism and low self-esteem were associated with subsequent development of psychosis or psychosis-like symptoms at 3-year follow-up in a Dutch population sample [43]. If we take the continuum hypothesis of psychosis into consideration [44], it is not surprising to find the same pattern in a first episode patient (FEP) sample.

However, there are studies showing that patient's self-stigma tends to be most affected during the early course of the disease, and that self-stigma and self-esteem are closely related [45]. It may be that there are sub-groups within the psychosis spectrum that differ with regard to stability in self-esteem, and also differ in which factors, either long or short term, have the most impact on levels of self-esteem. These complex mechanisms need to be explored further in longitudinal studies.

Gender was a significant predictor of self-esteem in this FEP sample, with women having significantly lower levels of self-esteem than men even after correction for differences in levels of depression. A vast body of literature from the general population indicates a small but significant gender difference in the same direction [46]. There is surprisingly little research on gender differences regarding self-esteem in psychosis but the present study is supported by findings from the Danish Opus trial [47] and suggests that gender difference is a factor which warrants further investigation.

The present study has some limitations. This is a cross-sectional study where conclusions about directions of relationships cannot be ascertained, and where data on premorbid adjustment is necessarily gathered retrospectively. There may also be a recall bias regarding the scores for premorbid adjustment. To what degree self-esteem is affected before the development of psychosis is thus not possible to test directly using the current design. Furthermore, the present study, due to the limitations of the study protocol, did not allow for more sophisticated measures of self-esteem, such as the measurement of fluctuations in self-esteem, which would have been of interest in this group.

## 6. Conclusion

The current study revealed both a significant association between premorbid adjustment and self-esteem and between self-esteem and positive psychotic symptoms. Future studies of self-esteem should consider examining how self-esteem changes over the course of illness from the prodromal stage. It would also be of interest to explore factors such as stigma and metacognition and their relation to self-esteem. There are several factors that in further studies could be explored as possible mediators in this context. Women with psychotic disorders report a high prevalence of sexual trauma [48] which is a known risk factor for low self-esteem. In addition, women's self-esteem may be more affected by medication induced weight-gain than men.

It is our opinion that this study may have clinical implications. It is possible that psychotheraputic interventions, such as cognitive behavioral therapy, may increase self-esteem and lessen the likelihood of development of positive psychotic symptoms or decrease their severity. Furthermore, psychotherapeutic intervention may help patients to acquire a broader personal narrative which would benefit their self-esteem.

## 7. Competing interests

The authors declare that they have no competing interests.

## 8. Authors' contributions

KLR has made substantial contributions to conception and design, acquisition of data, analysis and interpretation of data and drafting the manuscript. JIR has made substantial contribution to conception and design, analysis and interpretation, drafting of the manuscript and have been revising it critically for important intellectual content. CFH has made substantial contributions to acquisition of data and have been involved in drafting and revision of the manuscript. EH has made substantial contributions to acquisition of data and have been involved in drafting and revision of the manuscript. OAA has made substantial contributions to conception and design, drafting the manuscript and have been revising it critically for important intellectual content. IM has made substantial contributions to conception and design, drafting of the manuscript and have been revising it critically for important intellectual content. All authors have given final approval of the version to be published.

## Pre-publication history

The pre-publication history for this paper can be accessed here:

http://www.biomedcentral.com/1471-244X/11/136/prepub
